# Effects of Froggy Mouth Appliance in Pediatric Patients with Atypical Swallowing: A Prospective Study

**DOI:** 10.3390/dj12040096

**Published:** 2024-04-04

**Authors:** Andrea Scribante, Maurizio Pascadopoli, Simone Gallo, Paola Gandini, Pietro Manzini, Giulia Fadani, Maria Francesca Sfondrini

**Affiliations:** 1 Unit of Orthodontics and Pediatric Dentistry, Section of Dentistry, Department of Clinical, Surgical, Diagnostic and Pediatric Sciences, University of Pavia, 27100 Pavia, Italy; simone.gallo02@universitadipavia.it (S.G.); francesca.sfondrini@unipv.it (M.F.S.); 2 Independent Researcher, 46100 Mantua, Italy

**Keywords:** open bite, growing patients, orthopedic treatment, orofacial growth, dentistry, orthodontics, froggy

## Abstract

Atypical swallowing has a high incidence in growing subjects. Orthopedic treatment with orthodontic appliances and speech therapy are the main approaches to this problem. The aim of this prospective study was to evaluate the changes in the dental arches induced by one year of treatment with the Froggy Mouth myofunctional appliance designed to correct atypical swallowing. In total, 16 patients with atypical swallowing were instructed to use the Froggy Mouth appliance. A digital intraoral impression was taken at baseline (T0). The Froggy Mouth appliance had to be used for 15 min/day throughout the treatment period. At the end of the first year of treatment (T1), another impression was taken with the same intraoral scanner. Digital casts of the T0 and T1 impressions were obtained using software and the two casts were superimposed to record the following measurements: upper intercanine distance, upper arch diameter, upper arch width, overbite and overjet. The data were statistically analyzed (significance threshold: *p* < 0.05). Student’s t-test was used to compare pre- and post-treatment measurements. Linear regressions were performed to assess the influence of arch width on anterior and posterior diameters. A significant increase was found for the upper arch diameters (*p* < 0.05), whereas no statistically significant difference was found for the incisor relationship (overjet/overbite) (*p* > 0.05). To date, the efficacy of this appliance has not been extensively studied. According to the present prospective study, the Froggy Mouth protocol could be a valuable method as a myofunctional therapy for atypical swallowing, but further studies are needed to confirm these preliminary results.

## 1. Introduction

The normal process of swallowing in adults is represented by the positioning of the tip of the tongue on the incisive papilla with contact of the dental arches. A different pattern of swallowing is atypical swallowing, in which the tongue is positioned between the dental arches during this process, or sometimes there is an improper vestibular thrust against the upper frontal teeth [1,2]. When this condition occurs, treatment should be aimed at eliminating the tongue interference that prevents the proper eruption of teeth and bone growth [3].

During the first few years of life, rudimentary swallowing is considered physiological. Subsequently, the transition between mixed and final dentition, neuromuscular development and novel feeding methods lead to a progressive adaptation of the swallowing pattern [2,4,5]. Between 3 and 7 years of age is the period of transition from infantile to adult swallowing. Atypical (infantile) swallowing may be diagnosed if infantile swallowing persists beyond the previous upper limit [6]. Poor oral habits, incorrect eating habits or pathological problems are generally considered to be the main causes of this condition [6,7].

In terms of clinical signs, lingual interposition, mental muscle contraction and lower lip interposition between the dental arches are all signs of atypical swallowing [4]. This condition can affect several functions including chewing, breathing, speech and posture [8,9,10]. It can also affect the facial profile and mimicry, resulting in the hypertonia of the orbicularis oris muscle and the chin [5,8]. Lip strength is associated with altered jaw movements, such as mandibular protrusion [9] and labial incompetence, while it is also critical for maintaining balance in the anterior teeth [10]. 

The association between atypical swallowing patterns and other physiological functions can be explicated by considering the engagement of muscles in the lips, face, tongue, pharynx, larynx, and esophagus in both respiratory and swallowing processes. These muscular components serve critical roles in maintaining airway patency, ensuring airway protection, and facilitating the propulsion of food boluses. Dysphagia, characterized by swallowing disorders, and its multifaceted etiologies primarily impact these muscular structures and the surrounding connective tissues, resulting in functional impairments such as compromised bolus propulsion and potential airway compromise. Behavioral therapeutic modalities frequently incorporate compensatory strategies, including postural adjustments, maneuvers aimed at enhancing airway protection and bolus clearance, and regimens comprising strengthening exercises [11]. 

It is also believed that a significant deficit in daily function and facial growth may be caused by deficits in lip strength [11]. Therefore, the treatment of atypical swallowing is necessary to eliminate the harmful interference of tongue thrusting and to achieve the harmonious growth of the maxillofacial region [5,9,12].

Several devices have been proposed to treat atypical swallowing, including functional devices such as the Bionator [13], Fraenkel [14], eruption guidance appliances, lingual spurs [15], fixed appliances [16], as well as speech therapy treatment [17] and myofunctional therapy (MFT) [10,18,19,20]. One of the latest devices, the Froggy Mouth (FM), proposed in 2016 [18], consists of a small removable device made of thermoplastic material that is placed between the lips and inhibits both sucking and swallowing, and stimulates lip contraction to keep the device stable [16]. The use of the device for 10–15 min per day is justified by the use of the subcortical pathway to build new neural circuits [1]. The device is, therefore, considered to be a myofunctional appliance as it prevents bilabial contact, forces the tongue into a correct position, stimulates muscular training and, ultimately, induces a new swallowing pattern. An advantage of the Froggy Mouth is that it can be prescribed to young children and does not require analogue impressions or digital scans for its manufacture.

The aim of the present study was to evaluate the effects of Froggy Mouth therapy in growing children on orthodontic measures, specifically the upper intercanine distance, upper arch diameter, overbite, overjet and upper arch width after one year of treatment. The null hypothesis of the study was that there was no statistically significant difference when comparing the above outcomes before and after the Froggy Mouth therapy.

## 2. Materials and Methods

### 2.1. Study Design

This was a single-arm, prospective study conducted in accordance with the Declaration of Helsinki on experimentations involving human subjects and received approval by the Unit Internal Review Board (registration n: 2021-0512). The parents of the patients signed informed consent prior to the start of the study. The study started in June 2021 and ended in January 2023.

### 2.2. Participants

Pediatric patients attending the private practice of Dr. Pietro Manzini, 46100 Mantua, Italy, for oral care requiring orthodontic evaluation were enrolled in the study. The inclusion criteria were as follows:

- Age 5–12 years;
- No previous orthopedic and/or orthodontic treatment;
- No current orthodopedic and/or orthodontic treatment;
- Atypical swallowing diagnosed with lingual interposition between the dental arches during swallowing with contraction of the perioral muscles.

The following exclusion criteria were applied:

- Patients receiving speech therapy for atypical swallowing;
- Previous speech therapy for atypical swallowing.

### 2.3. Interventions and Outcomes

**Figure 1:**
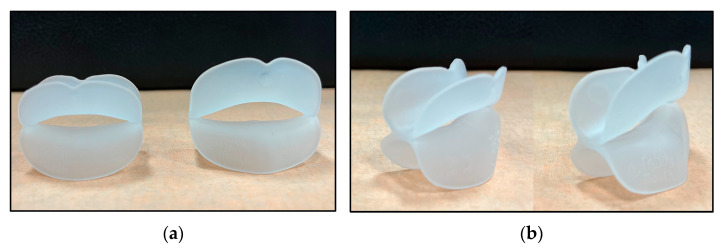
Froggy Mouth appliance (S size on the left and M size on the right): (**a**) frontal vision; (**b**) lateral vision.

**Figure 2:**
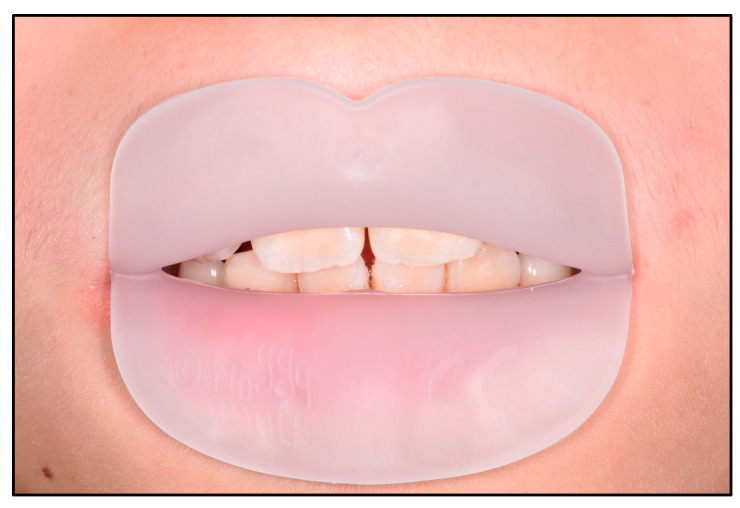
Patient wearing Froggy Mouth appliance.

Patients were visited and parents were proposed to study the orthodontic case. Intraoral and extraoral photographs, digital impressions, orthopantomography and lateral cephalometric radiograph were performed for each patient. The case was discussed with parents and acceptance of the treatment plan was provided. At the beginning of the study (baseline, T0), patients were instructed to use the commercially available Froggy Mouth appliance (ATFC srl, Alpago, BL, Italy) (Figure 1), a small removable appliance created by a thermoplastic elastomer. The appliance is available in different sizes, and the clinician chose the right size for the patients. Children were subdued again to an impression taken with an intraoral scanner (3Shape TRIOS 3, 3Shape, Copenhagen, Denmark). The Froggy Mouth appliance had to be used for 15 min/day throughout the treatment period (Figure 2). No specific exercises or modifications were recommended while wearing the appliance.

Patients were visited every two months to evaluate their comfort and compliance with the treatment that was monitored by asking both the patients and the parents if the appliance was worn.

**Figure 3:**
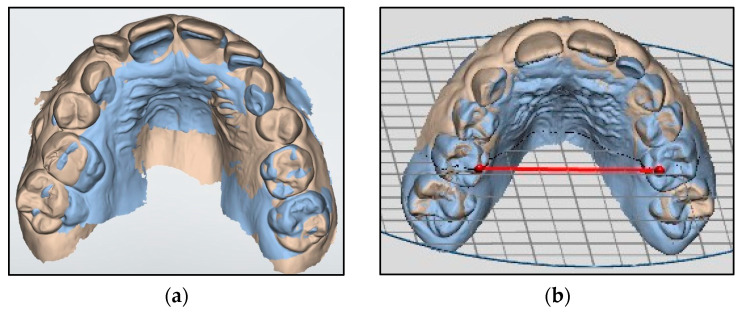
Digital models from intraoral impressions: (**a**) superimpositions of pretreatment models (blue) and post-treatment models (brown), (**b**) section of upper arch at the level of second deciduous molars.

**Figure 4:**
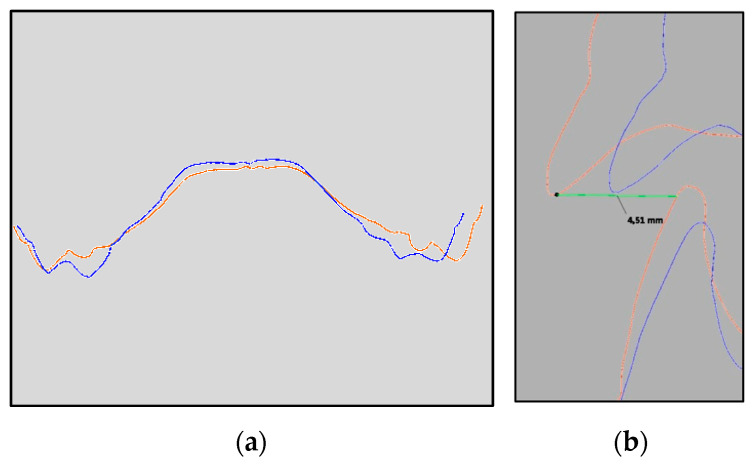
Measures performed on the digital casts: (**a**) results of the section at the level of second deciduous molars, (**b**) section at the incisor level for OJ measurement. Legend: blue line, pretreatment cast; red line, post-treatment cast.

At the end of the first year of treatment (T1), the patient was re-evaluated, and another impression was taken with the same intraoral scanner. The digital casts of T0 and T1 impressions were elaborated with 3Shape OrthoAnalyzer software (version 1.9.3.2, 3Shape) and the superimposition (Figure 3) of the two casts was performed with the aim of obtaining the following measures:

- Upper intercanine distance: distance between the edge of the cusps of the deciduous canines;
- Upper arch diameter (Figure 4a): distance between the mesio-palatal cusps of upper second deciduous molars;
- Upper arch width: distance between upper interincisal point and the point of intersection between the straight line passing from the furthest point from the crown of the second deciduous molars and the perpendicular line passing from the interincisal point;
- Overbite: distance between the uppermost vertically erupted middle incisor and the corresponding incisal edge of the opposite mandibular tooth;
- Overjet (Figure 4b): distance between the most palatal point of the maxillary central incisors and the corresponding reference point on the vestibular surface of the mandibular incisor.

### 2.4. Sample Size

Type I error (alpha) = 0.05 and type II error (power) = 80% were set to calculate the sample size of the study for the chosen primary outcome “intercanine distance”. The calculation was based on the results of Garg and colleagues [19], hypothesizing an expected value of 51.4, an expected mean of 1.27 and a standard deviation of 1.28, requiring 16 patients for the enrollment. 

### 2.5. Statistical Analysis

Data were statistically analyzed using R software (R version 3.1.3, R Development Core Team, R Foundation for Statistical Computing, Wien, Austria). The Shapiro–Wilk test was performed, and it revealed the normal distribution of data for all the variables. For each group and variable, descriptive statistics, including mean, standard deviation, median, minimum, maximum, and Cohen’s d standardized effect size, were measured for each group. The Student’s *t* test was performed for all the variables tested. Pearson’s correlation coefficients were calculated to assess the mutual influence of the variation in the upper arch width, upper intercanine distance, and upper arch diameter.

Statistical significance was set at *p* < 0.05 for all statistical tests.

## 3. Results

### 3.1. Demographic Data

**Figure 5:**
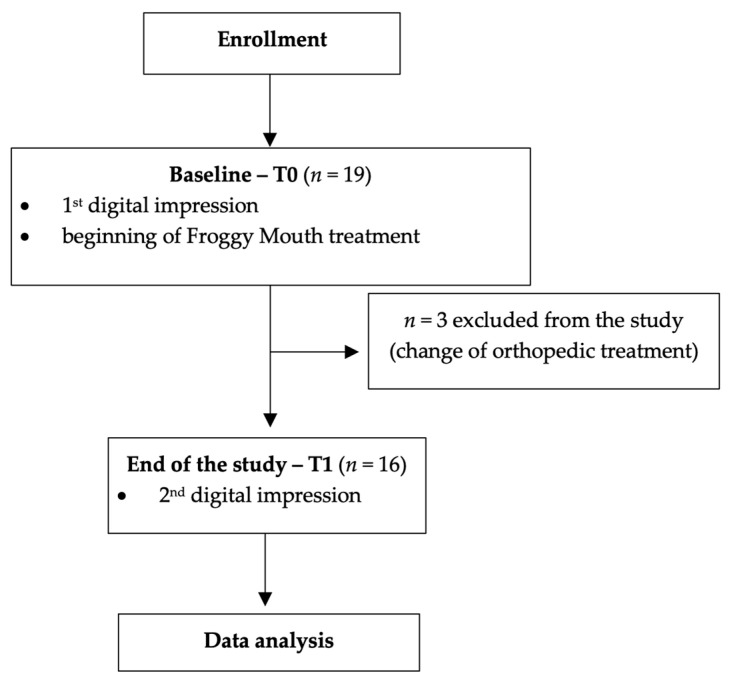
Flowchart of the study. Legend: *n*, number of patients.

A flowchart of the randomized clinical trials from enrollment to data analysis is shown in Figure 5. Initially, 19 patients were included in the study. During the conduct of the study conduction, 3 patients had to be excluded because they were scheduled for a second phase of treatment with different orthotic devices. Enrolment continued until the sample size was reached. Finally, 16 patients were analyzed at the end of the study. Their mean age was 9.06 ± 1.29 years, 8 males (8.96 ± 1.46 years) and 8 females (9.15 ± 1.21). 

### 3.2. Statistical Analysis Results

In terms of the variables tested (Table 1), statistical significance was achieved after one year of treatment for upper intercanine distance (*p* = 0.04), upper arch diameter (*p* = 0.02) and upper arch width (*p* = 0.01). However, the T0-T1 difference was not significant for overbite (*p* = 0.43) and overjet (*p* = 0.77) measurements.

Pearson’s correlation coefficients (Table 2) used to assess the mutual influence of the variation in the upper arch width, upper intercanine distance, and upper arch diameter resulted in weak correlations.

## 4. Discussion

Orthopedic treatment in growing patients is desirable to correct skeletal discrepancies before aligning teeth. Various research has been performed to assess the best timing for intervention, considering the influence of factors like sex, age and compliance of the patients [20]. Generally, the first problem that should be corrected is maxillary transverse discrepancy [21], while secondary interventions should involve sagittal discrepancies’ correction, with an approach involving functional appliances [22]. 

Atypical swallowing is an alteration in the correct pattern of deglutition that usually occurs in the early stages of growth and evolves towards correct swallowing with tooth eruption [23]. In fact, infantile swallowing consists of moving the tip of the tongue forward. This type of swallowing can persist beyond the fourth year of life and can be considered an atypical mechanism, often associated with other anomalies such as anterior open bite (AOB) and increased overjet [6]. Tongue thrust can, therefore, be considered in two types of patients; namely, younger children, as a physiological stage of swallowing maturation, and patients of any age, due to predisposing factors such as increased overjet and anterior open bite, which facilitate the forward position of the tongue. Intercepting tongue thrust in the early years of life is surely easier than approaching the problem in later stages [24]. 

The null hypothesis of the study was partially rejected. In fact, the present study showed that after one year of treatment, the Froggy Mouth device caused a significant increase in the upper intercanine distance, the upper arch diameter and the upper arch width, whereas no effect was found on the parameters reflecting the relationship between the central upper and lower incisors (i.e., overjet and overbite). To date, no comprehensive studies have been conducted on the Froggy Mouth device and, to the best of our knowledge, only three studies have been published in the literature examining the effects of this device on orthodontic outcomes: two prospective studies and a case series. Di Vecchio and colleagues [18] tested the Froggy Mouth device on 370 patients to evaluate its influence after 9–12 years of treatment. The study consisted of the collection of intraoral and extraoral photographs of the patients, impressions, orthopantomographies and lateral cephalograms, as well as clinical records and other information obtained through a questionnaire. According to the results obtained, the authors found that the appliance showed positive clinical results in resolving malocclusions in growing patients, including open bite, transverse palatal constriction, cross bite and deep bite. Froggy Mouth also helped to resolve other problems such as snoring, drooling, sleep apnoea and difficulty breathing through the nose. Extraoral photographs showed the change in the face, neck and shoulder position immediately after wearing the device, suggesting a direct correlation between the altered contraction of the perioral and masticatory musculature and the asymmetrical contraction of the cervical musculature, with implications for TMJ and posture. Manzini et al. [25] reported the clinical use of the Froggy Mouth appliance in a case series; however, no measurements or comparisons of clinical studies were performed.

A prospective study was also carried out by Quinzi and colleagues [26], who evaluated the effect of the functional device on atypical swallowing, assessing lip force and altered facial mimicry. The benefits of the appliance were evaluated in 40 children with atypical swallowing before and during 6 months of treatment. The study showed that after 6 months of treatment, 82.5% of the subjects showed good compliance and all achieved a corrected swallowing pattern. Of these, 2 children (5% of the total sample) achieved early correction after only 3 months, 5 children (12.5% of the total sample) after 4 months, 11 children (27.5% of the total sample) after 5 months and 15 children (37.5% of the total sample) after 6 months. In total, 17.5% of the total sample (7 children out of 40) did not adequately follow the recommended protocol and refused the device; thus, not achieving the expected result. In patients exhibiting high compliance, treatment success was achieved in all cases. Clinical observations showed that most patients progressed in a steady manner throughout the observation period and eventually achieved the result. Therefore, the authors concluded by highlighting the short-term efficacy of this myofunctional appliance in the treatment of atypical swallowing, achieving the correction of facial mimics and labial incompetence with a significant improvement in lip strength.

The current research has mainly focused on the effectiveness of the Froggy Mouth device on the diameter of the maxillary arch and on the relationship between the incisors (overjet/overbite), whereas no direct evaluation was performed to test the effect on atypical swallowing. On the basis of these considerations, the results of the present study can be partially compared with those of the authors mentioned above. However, even Di Vecchio et al. [18] concluded that Froggy Mouth showed positive clinical results in resolving malocclusions in growing patients, such as transverse palatal constriction, but they also found an improvement in deep bite, whereas no effect on overbite and overjet was assessed in the present study. Conversely, Quinzi et al. [26] evaluated parameters directly related to atypical swallowing, but not to upper arch diameter and incisor ratio; thus, not allowing comparison with the current research. Certainly, atypical swallowing and tongue thrust can be adopted as criteria for using this orthopedic appliance, considering the fact that patient compliance is fundamental. The first phase of treatment can be faced with this appliance and then it can be continued with other compliance-related appliances [27]. However, the biggest disadvantage of the Froggy Mouth appliance is probably the fact that it is a removable appliance; therefore, the results of the treatment depend on patients’ compliance. In the present study, a mean improvement in arch parameters was noted, but, surely, other variables should be considered for further research. The main limitation of this study is that it was a prospective protocol without a control group to compare with the experimental group. The lack of a control group with no treatment is justified by ethical reasons; however, it could be considered worthwhile to add an active comparison with other functional appliances. Additionally, it could be considered beneficial to add patients that only attend the dental setting without undergoing orthopedic treatment due to parental choice, to assess in an ethical way the natural growth versus orthopedic treatment results. Furthermore, only orthodontic parameters were assessed, without including the results of atypical swallowing and perioral muscle activity. The one-year follow-up could not be enough to evaluate long-term dental changes and potential relapse. Therefore, future randomized controlled trials should be performed to better clarify the effect of the Froggy Mouth device on both orthodontic malocclusion and atypical swallowing for longer durations of the studies and with active controls. Specifically, other populations and other clinical variables should be considered and comparisons of this treatment protocol with other devices and with logopedic therapy, alone or in combination, should be considered [4,28]. Additionally, the correlation between sleeping disorders and anterior open bite, together with myofunctional treatments, should be furtherly explored [29]. Patients’ perceptions during therapy and the feasibility of use of the appliance could be further evaluated. Considerations on adult patients should be performed, suggesting the use of the Froggy Mouth appliance in light of the fact that tongue thrust can cause severe open bite even in patients with post-orthodontic retention appliances like fixed multibraided retainers [30].

## 5. Conclusions

The clinical protocol based on the use of the Froggy Mouth appears to be effective in atypical swallowing patients for improving upper arch diameters, including upper intercanine distance, upper arch diameter and upper arch width, while no significant changes were found for overbite and overjet measurements. Further scientific studies with the addition of control groups are needed to support these early results and explore other clinical variables.

## Figures and Tables

**Table 1:** Descriptive statistics of the variables assessed in the study. * Statistical significance was set for *p* < 0.05.

Group	Time	Mean	SD	Min	Median	Max	SES	Significance
Upper intercanine distance	T0	31.86	2.54	27.60	32.25	36.18		
	T1	32.78	2.19	27.70	32.83	35.66		
	T1-T0						0.388	0.042 *
Upper arch diameter	T0	35.02	2.35	28.90	35.11	38.53		
	T1	36.07	2.05	31.75	36.22	39.33		
	T1-T0						0.476	0.016 *
Upper arch width	T0	30.25	3.62	24.62	29.27	40.00		
	T1	31.96	3.35	28.04	31.58	42.00		
	T1-T0						0.490	0.012 *
Overbite	T0	1.08	1.49	−1.66	1.09	3.38		
	T1	1.26	1.61	−2.70	1.59	3.66		
	T1-T0						0.116	0.433
Overjet	T0	3.57	2.46	−2.16	4.05	7.43		
	T1	3.69	1.49	0.42	3.42	7.40		
	T1-T0						0.048	0.769

Legend: SD, standard deviation; min, minimum; max, maximum; SES, Cohen’s d standardized effect size.

**Table 2:** Pearson’s correlation coefficients (r) of the variations in the upper intercanine distance, upper arch diameter, and upper arch width.

Variables	r
∆ Upper intercanine distance~∆ Upper arch diameter	−0.0783
∆ Upper intercanine distance~∆ Upper arch width	0.2382
∆ Upper arch diameter~∆ Upper arch width	−0.1578

## Data Availability

Data are available upon motivated request to the corresponding authors.
